# Hospitalization for diseases attributable to human papillomavirus in the Veneto Region (North-East Italy)

**DOI:** 10.1186/1471-2334-13-462

**Published:** 2013-10-05

**Authors:** Vincenzo Baldo, Silvia Cocchio, Alessandra Buja, Tatjana Baldovin, Patrizia Furlan, Chiara Bertoncello, Mario Saia

**Affiliations:** 1 Department of Molecular Medicine, Public Health Section, Istituto di Igiene, University of Padua, Via Loredan, 18, 35121 Padova, Italy; 2 EuroHealth Net, Veneto Region Health Directorate, Venezia, Italy

## Abstract

**Background:**

Human papillomavirus (HPV) is one of the most common sexually-transmitted pathogens. A number of studies in the literature have estimated the burden of HPV-related diseases by collecting data at primary care level, while a comprehensive assessment of the global burden of HPV-related diseases on hospital resources is still lacking.

**Methods:**

This was a retrospective cohort study based on hospital discharge data collected from 2000 to 2010 in the Veneto Region (north-east Italy). All hospitalizations for diseases potentially associated with HPV were identified by searching the hospital discharge records, then the proportion of these hospitalizations relating to diseases attributable to the HPV infection was calculated.

**Results:**

Overall, the annual hospitalization rate for HPV-related diseases was 21.3 per 100,000 individuals in the general population, 15.8 per 100,000 males, and 27.6 per 100,000 females. Hospitalizations were due mainly to genital warts, and peak among 15- to 44-year-olds in both genders. Taking both sexes together, the hospitalizations attributable to HPV dropped from 24.5/100,000 in 2000 to 17.5/100,000 in 2011, showing a significant decline during this period, with an average annual percent change (AAPC) of −1.9% (CI 95%: -2.8, -0.9;). On the other hand, the hospitalization rate for genital warts tended to increase significantly (AAPC 3.0% [CI 95%: 1.4;4.7]), whilst there was a significantly declining trend in the hospitalization rate for anal cancer (AAPC - 5.0% [CI 95%: -7.7;-2.2]), genital cancer (AAPC −6.2% [CI 95%: -7.8;-4.6]) and oropharyngeal cancer (AAPC −4.3% [CI 95%: -4.8;-3,8]).

**Conclusion:**

Data derived from the hospital records indicate that HPV-related diseases are an important public health issue.

## Background

Human papillomavirus (HPV) is the most common sexually-transmitted pathogen [1]. In the USA, more than one in two sexually-active individuals become infected with one or more types of HPV at some point in their lives. Recent research indicates that, at some point in time, 42.5% of women have had genital HPV infections, whereas less than 7% of adults have had oral HPV infections [2,3]. The estimated overall epidemiological burden of HPV-related cancers and non-malignant diseases is very high in Europe too [4].

Most infections are asymptomatic and transient, but certain HPV types are oncogenic. Numerous studies have identified many biochemical features that contribute to HPV-induced carcinogenesis, such as the ability of the viral proteins E6 and E7 to bind cellular factors, and biological properties like the capacity of HPV proteins to induce cell immortalization [5].

Genital infection with oncogenic or high-risk types of HPV is a causal factor in cervical cancer; the HPV types mainly involved are 16 and 18, which are responsible for approximately 70% of cases of this cancer. HPV also has a well-known role in premalignant lesions of the cervix, vulva and vagina, and the International Agency for Research on Cancer (IARC) has recently supported (with “sufficient” or “limited” evidence) the carcinogenicity of HPV in the penis, anus, oral cavity, oropharynx, tonsils and larynx [6]. A recent review found that HPV was considered responsible for about 88% of anal cancers in both sexes, 70% of vaginal cancers, 43% of vulvar cancers and 50% of penile cancers [7]. Many authors have highlighted the role of HPV in cancers involving the oropharynx and oral cavity too [8]. In 2005 Kreimer et al. conducted a systematic meta-analysis on the available literature on the topic and ascertained the worldwide prevalence of HPV in neck squamous cell carcinoma, which was estimated at 26% [9].

HPV is responsible for several benign diseases as well, e.g. genital warts, that have a significant economic and psychological impact [10,11]. Genital warts are the most commonly diagnosed disease among males referred to centers for the treatment of sexually-transmitted diseases [8].

A number of studies in the literature have estimated the burden of HPV-related diseases by collecting data at primary care level, while only one European study (conducted in Spain) used the hospitalization records to ascertain the burden of HPV related to anal and penile neoplasms [12]. No comprehensive assessments have been conducted on the overall burden of HPV-related diseases on hospital resources. Though by no means the only burden of HPV-related diseases, the related hospitalization costs are certainly the most important in economic terms.

The aim of the present study was to estimate the burden of hospitalization for HPV-related diseases, stratified by sex, in a population of almost five million citizens.

## Methods

To estimate the annual HPV-associated hospitalization rates, we analyzed the data in the hospital records from 2000 to 2011 in the Veneto Region (north-east Italy) concerning all discharges from all public and accredited private hospitals, including day hospital services. HPV-associated hospitalization were identified by selecting all the hospital discharge records containing one of the following codes in the International Classification of Diseases, Ninth Revision, Clinical Modification (ICD-9-CM): genital warts; 078.11 “Condyloma acuminatum”; anal cancer; 154.2-154.8 “Malignant neoplasm of anus”; oropharyngeal cancer; 146.0-146.9 “Malignant neoplasm of oropharynx”; 171.0 “Malignant neoplasm of head, face, and neck”; genital cancer; 187.1-187.9 “Malignant neoplasm of penis”; 180.0-180.9 “Malignant neoplasm of cervix uteri”.

Only admissions for which HPV-related diseases were indicated as the main diagnosis at the time of discharge, were drawn from the database. The proportion of hospitalizations for diseases potentially associated with HPV infection was calculated on the assumption that all discharges for cervical cancer and genital warts were HPV-related, while this was true of 50% and 88%, respectively, of cancers of the male genitalia (penis) and anus, and applied to 26% of oropharyngeal cancers [7]–[9]. The HPV-related hospitalization rate was calculated from the number of discharges as a proportion of the Veneto’s resident population in a given year.

Significant trends over the years considered were assessed as average annual percent changes (AAPC), a summary measure of the trend over a given fixed interval. It is computed as a weighted average of the annual percent change (APC) emerging from the join-point model, using weights equating to the length of the APC interval. If an AAPC lies entirely within a single join-point segment, the AAPC is the same as the APC for that segment [13].

Data were obtained from the Regional Statistics Office and analyzed using EPI-Info 2000 software (Center for Disease Control and Prevention of Atlanta, GA, USA) and the Joinpoint Regression Program, rel. 4.0.4. May 2013 (Statistical Research and Applications Branch, National Cancer Institute, USA).

The study was conducted on data routinely collected by the health services linked to anonymized records that make it impossible to identify the individuals concerned. The data analysis was performed on the anonymized aggregated data. Data in the Local Health Authority registries are recorded with the patient’s consent and can be used as aggregated data for scientific studies without further authorization [14]. This study complies with the Declaration of Helsinki and with Italian privacy law (Decree n. 196/2003 on the protection of personal data).

## Results

We identified 16,659 hospitalizations for diseases potentially associated with HPV between January 1, 2000 and December 31, 2011. The proportion of these hospital admissions attributable to HPV was calculated to be 12,410 (74.5% of the total), comprising 4,384 males (35.3%) and 8,026 females (64.7%). Genital warts were the most common HPV-related condition prompting admission to hospital (44.9%).

Genital warts were usually associated with age under 45 years (4,613; 82.8%), while the HPV-related cancers were more common in patients aged ≥ 45 years (5,728; 83.7%). Table 1 shows the HPV-related admissions by sex and age group.

**Table 1:** Number of HPV-related hospitalizations in the Veneto Region (2000–2011)

	**Total**	**Genital warts**		**Genital cancer**		**Oropharyngeal cancer**		**Anal cancer**	
		**n**	**(%)**	**n**	**(%)**	**n**	**(%)**	**n**	**(%)**
**Total**	12,410	5,570	(44.9)	3,905	(31.5)	1,258	(10.1)	1,677	(13.5)
**Gender**									
males	4,384	2,097	(47.8)	438	(10.0)	1,000	(22.8)	849	(19.4)
females	8,026	3,473	(43.3)	3,467	(43.2)	258	(3.2)	828	(10.3)
**Age group**									
<14	44	30	(68.2)	2	(4.5)	10	(22.7)	2	(4.5)
15-24	1,230	1,211	(98.5)	12	(0.9)	5	(0.4)	2	(0.2)
25-44	4,451	3,372	(75.8)	951	(21.4)	51	(1.1)	77	(1.7)
45-64	3,657	811	(22.2)	1,621	(44.3)	666	(18.2)	559	(15.3)
65-74	1,544	112	(7.3)	643	(41.6)	339	(22.0)	450	(29.1)
75-84	1,154	31	(2.7)	533	(46.2)	154	(13.3)	436	(37.8)
85+	331	3	(0.9)	144	(43.4)	33	(10.0)	151	(45.7)

Overall, the average hospitalization rate for HPV-related diseases in the years 2000–2011 was 21.8 per 100,000 (15.8 per 100,000 males, and 27.6 per 100,000 females). Table 2 shows the average hospitalization rate for HPV-related diseases in 2000–2011 per 100,000 population in the Veneto Region by type of disease and gender.

**Table 2:** Average hospitalization rate in 2000–2011 for HPV-related diseases per 100,000 population in the Veneto Region, by disease and gender

	**Males**	**Females**	**Total**
**Genital warts**	7.5	11.9	9.8
**Genital cancer**	1.6	11.9	6.9
**Anal cancer**	3.1	2.8	2.9
**Oropharyngeal cancer**	3.6	0.9	2.2
**Total**	15.8	27.6	21.8

Considering both sexes together, the hospitalization rate attributable to HPV dropped from 24.5/100,000 in 2000 to 17.5/100,000 in 2011; this decline was statistically significant, with an AAPC of −1.9% (CI 95%: -2.8, -0.9;) from 2000 to 2011. On the other hand, the trend of the hospitalization rate for genital warts increased significantly (AAPC 3.0% [CI 95%: 1.4;4.7]), whilst there was a significantly declining trend in the hospitalization rate for anal cancer (AAPC - 5.0% [CI 95%: -7.7;-2.2]), genital cancer (AAPC −6.2% [CI 95%: -7.8;-4.6]), and oropharyngeal cancer (AAPC −4,3% [CI 95%: -4.8;-3.8]).

Over the whole study period, the HPV-attributable hospitalization rate for males remained stable, going from 16.1/100,000 in 2000 to 14.6/100,000 in 2011 (AAPC 0.2% [CI 95%: -0,8;1,2]), while for females it decreased from 32.5/100,000 in 2000 to 20.2/100,000 in 2011 (AAPC −2.9% [CI 95%: -4.2; -1.6]).

**Figure 1:**
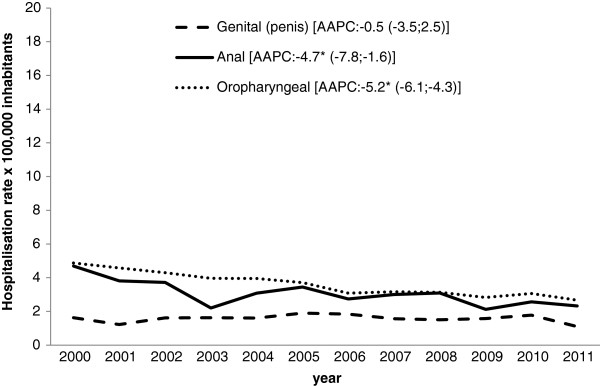
Hospitalization rate (× 100,000 population) for HPV-related cancer in males in the Veneto Region (2000–2011), by type of cancer. * p<0.05.

**Figure 2:**
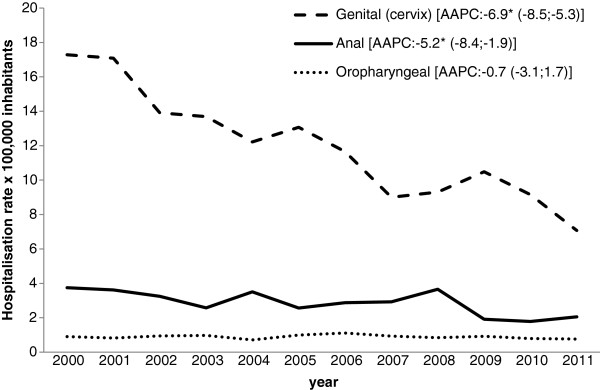
**Hospitalization rate (× 100,000 population) for HPV-related cancer in females in the Veneto Region (2000–2011), by type of cancer.** * p<0.05.

Looking at the admissions for HPV-related cancer in males, the hospitalization rate decreased significantly for anal cancer (AAPC −4.7% [CI 95%: -7.8; -1.6]) and oropharyngeal cancers (AAPC −5.2% [CI 95%: -6.1; -4.3]), while for genital (penis) cancer it remained stable (AAPC −0.5% [CI 95%: -3.5; 2.5]) (Figure 1). As for the admissions for HPV-related cancer in females, there was a significant drop in the figures for genital (cervix) cancer (AAPC −6.9% [CI 95%: -8.5; -5.3]) and anal cancer (AAPC −5.2% [CI 95%: -8.4; -1.9]). For oropharyngeal cancer, in female, the hospitalization rates did not change significantly over the study period (AAPC −0.7% [CI 95%: -3.1; 1.7]) (Figure 2).

**Figure 3:**
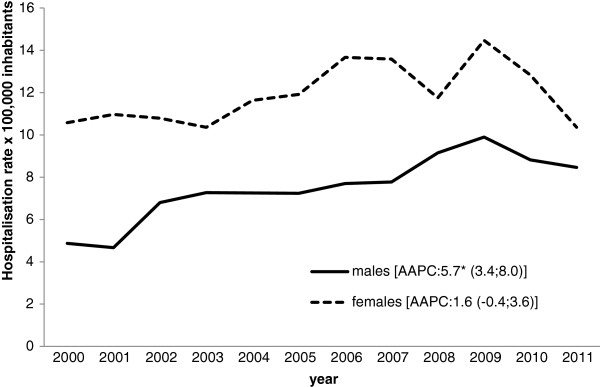
**Hospitalization rate (× 100,000 population) for genital warts in the Veneto Region (2000–2011) by gender.** * p<0.05.

There was a statistically significant increase over the years in the hospitalization rate for genital warts in males (AAPC 5.7% [CI 95%: 3.4;8,0]), but not in females (AAPC 1.6% [CI 95%: -0.4; 3.6]) (Figure 3). For these non-malignant conditions, there was a female predominance in all age groups, with a female-to-male ratio of 1.8:1.

**Figure 4:**
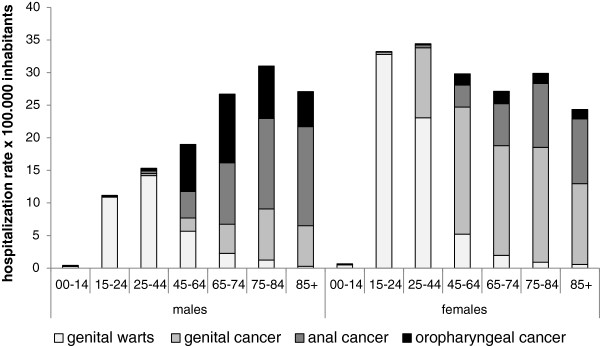
HPV-related hospitalization rates (× 100,000 population) in the Veneto Region by gender, type of disease, and age group (2000–2011).

Figure 4 shows the hospitalization rates for HPV-related non-malignant diseases by age group and gender. The highest hospitalization rate was for genital warts in 15- to 44 year-olds of both genders. Hospitalization for HPV-related cancer increased with age and the female-to-male ratios were 7.9:1, 1:1, and 1:3.9 for genital, anal and oropharyngeal cancers, respectively.

## Discussion and conclusions

The present study examined the trends in the hospitalization rates for different types of HPV-related disease in a cohort of 5 million citizens and the results show that the burden of HPV-related diseases on hospital resources is an important public health issue.

It is not easy to estimate the morbidity of HPV-related diseases in the general population, partly because the currently-available epidemiological picture is hazy due to differences in study designs and population profiles, access to health care and registration methods (especially for benign diseases). Hospital discharge data can, however, be useful for assessing the cases severe enough to warrant hospitalization [15].

Using discharge diagnoses to identify cases of HPV-related diseases is more useful for cancer than for benign diseases. Patients are only hospitalized for genital warts in the more complex cases, and particularly for those requiring surgical removal [16].

The overall hospitalization rate identified was 21.8/100,000 population during the calendar years 2000–2011, and it was 1.8 times higher for females than for males. This might suggest a gender-related difference in the natural history of HPV, with lower infection and higher disease rates in women, and vice versa in men [17,18]. The age groups considered in our sample revealed age-related differences in the hospitalization rates for the diseases examined: more older people had cancer and more younger people had condylomatosis. This picture may be due to the age groups’ different times of exposure to HPV, and to different latency periods of the various diseases [1]. Another significant aspect concerns the hospitalization rate for genital warts, which accounted for almost one in five of all HPV-related hospital stays. For this disease, the hospitalization rate peaked for men slightly later in life than for women (at 25–44 years of age in the former and 15–24 in the latter). Our figures are similar to those reported in North American and Australian studies [18], whereas the peak age was the same for the two genders in the United Kingdom and Nordic countries [19,20].

Our data show that a drop in the hospitalization rates for women from 2000 to 2011. This concerns the hospitalization for cancer, and cancer of the uterine cervix in particular, and relates to the introduction of organized screening programs for detecting and treating this disease in our region. In accordance with Italian national guidelines, cervical cancer screening programs have been implemented in the Veneto Region since 1997. They are based largely on European guidelines and women from 25 to 64 years of age are personally invited for a Pap test every three years [21].

There was also a smaller drop in the hospitalization rate for anal cancer, that was statistically significant in males. A possible explanation for this could lie in changes in the management of chemotherapy and radiotherapy for cancer: since 2007 they have been administered not in hospital but at ambulatory outpatient services [22].

The overall trend of our HPV-related hospitalizations nonetheless revealed an increase among males during the study period. The hospitalization rates rose for genital warts in males during the period considered (pointing to the need for prevention programs), but not in females. A higher prevalence among males of severe HPV-related warts warranting hospitalization could be expected to induce a corresponding increase in women too, given the transmissible nature of HPV [23], but this was not the case - probably because the early diagnosis of warts on cervical cancer screening prevented them from growing enough to need hospitalization.

This study shows that hospitalization for genital warts has a considerable impact on the health services in the Veneto Region. Vaccination strategies can be used for the primary prevention of HPV infection, and could reduce the burden of HPV-related disease. Two vaccines against HPV have been developed and are currently used in population vaccination programs in many countries. Both have proved highly effective in preventing HPV 16 and 18, which are jointly responsible for approximately 70% of cervical cancers [24]. A quadrivalent vaccine (approved for use in males and females aged 9–26 years) also protects against the HPV types responsible for 90% of genital warts (types 6 and 11) [25]. The economic burden of HPV-related conditions on hospital resources should be taken into account when assessing the potential benefits of HPV vaccines against HPV 6 and 11, since it is expected that these vaccines will ultimately reduce the incidence of HPV-related diseases.

Presently there has been ongoing debate over the introduction of HPV vaccination. Before introducing the vaccination in males should be considered the vaccination coverage rate in females, the burden of HPV-related diseases and whether it is programmatically feasible and economically sustainable.

In Italy, since 2007, free anti-HPV vaccination has been offered to all girls in their 12th year of life. Two different vaccines have been available in Italy for the past three years: a bivalent vaccine directed against HPV types 16 and 18 (involved in cancer of the uterine cervix and other cancers) and a quadrivalent vaccine against HPV 16 and 18, and 6 and 11 (the latter are responsible for the onset of external genital lesions, i.e. condylomas and genital warts, which can affect both genders) [7,8]. A recently-published Italian progress report on the vaccination programs identified a mean coverage of 70.6%, 69.7% and 69.3%, respectively, in the 1997, 1998 and 1999 cohorts for the first dose, while the full vaccination cycle was completed in 66.0%, 64.0% and 58.6% of cases, respectively. In the Veneto Region, the coverage was 76.8%, 73.6% and 61.3%, respectively, for the female cohorts in 1997, 1998 and 1999 [26]. As HPV is sexually transmitted, males play an important part in the pathology of HPV, not only as transmission agents, but also as targets of HPV infection, so the universal vaccination of adolescents would limit its transmission and significantly reduce the virus’s circulation in the general population. It could also have the advantage of reducing the rate of HPV-related disease among males, which is far from negligible, as the present study demonstrates. The high proportions of HPV-positive cancers attributable to HPV16/18, and of non-malignant diseases attributable to HPV6/11 underscore the potential importance of preventing the majority of male HPV-related diseases by means of prophylactic vaccination [27].

Our study has some limitations. Among them, we should consider the dubious accuracy of the total number of HPV-related hospital admissions identified due to coding errors in the hospital records. Another limit lies in that the responsibility of HPV could not be tested because no virology data were available. Judging from our hospitalization records, it would seem important to conduct prospective epidemiological studies with the cooperation of specialists in this field, with a view to obtaining a more precise picture of the epidemiological situation regarding HPV-related diseases.

## Competing interests

The authors declare that they have no competing interest.

## Authors’ contributions

VB: data analysis, supervision, drafting of the manuscript; FP statistical expertise, drafting of the manuscript CS: study conception and design, drafting of the manuscript; MS: study conception and design, data collection; TB: study conception, supervision; CB: intellectual content, drafting of the manuscript; AB: statistical expertise, data interpretation and supervision. All authors read and approved the final manuscript.

## Pre-publication history

The pre-publication history for this paper can be accessed here:

http://www.biomedcentral.com/1471-2334/13/462/prepub
